# Buttermilk in Sick Stomach

**Published:** 1885-07-20

**Authors:** R. J. Pere

**Affiliations:** Pleasanton, Kan.


### BUTTERMILK IN SICK STOMACH.

BY R. J. PERE, M.D., PLEASANTON, KAN.

An irritable stomach, it will be admitted, is often a most serious complication in the management of sickness. In occasional cases, of no particular gravity otherwise, oftenest in diseases of children, this difficulty leads to a fatal issue. Buttermilk, so far as I am aware, is an untried remedy in such cases. I have had some experience recently with it, quite satisfactory in a few instances. Four cases of persistent vomiting occurring in succession, intolerant of any other treatment, gave way kindly to this.

### CASE I.

Was that of a child about two years old. The vomiting was unaccompanied by other sickness. The child had not retained anything, fluid or solid, for two days; the food being almost immediately ejected. I suggested buttermilk in teaspoonful quantities, every ten, then every five, minutes, the milk to be quite cold and as fresh as possible. The vomiting did not recur, and in two days the child had changed from a condition of impending death, from collapse, to nearly its normal condition. In place of teaspoonful quantities, the stomach soon sustained larger ones, and so on till an ordinary quantity could be taken.

### CASE II.

Was that of a nursing child suffering from a mild derangement of the digestive process, accompanied by fever and persistent vomiting while anything remained in the stomach. The mother's milk was immediately rejected. I again ordered buttermilk, in the same manner as before, much to the surprise of the parents. Next day the father reported that there had been no vomiting from the time this treatment was commenced.

### CASE III.

This was an adult female. Three weeks before she had been confined, and at this time was suffering from a mild attack of peritonitis, with constipation and nervous nausea in this case, even when the stomach was empty—a feature in which it differed from the other three. Buttermilk was cooled with ice, and carefully given in gradually increasing quantities till it was retained quite well, after other remedies had all failed, and in twelve hours it could be taken freely. The nausea was overcome with more difficulty in this case than in the others.

CASE IV.

Was that of a child one year old and weaned. The mother had been away from home some distance with the child, visiting. While absent a slight diarrhœa occurred, accompanied by sick stomach. When I saw it the stomach difficulty predominated greatly. Everything given was immediately expelled with force. The mildest remedies were not retained a moment. The stomach was intensely sour, and food taken therein days before was passed from the bowels undigested. Buttermilk, as directed in the other cases, was ordered, with lime-water. The vomiting subsided very quickly, and the stomach could soon tolerate boiled milk thickened with flour. This change became necessary on account of the condition of the bowels, which now became as intolerant of the buttermilk as the stomach had been, the milk passing through immediately after ingestion. After the change of food no passage occurred for twenty-four hours.

So far as I have observed, buttermilk does not coagulate in the stomach, as does new milk. This is, perhaps, its only advantage over the latter, but one of inestimable value, since the coagulation of new milk casein, so likely to occur, utterly forbids its use in many cases. In the "summer complaints" of children, for instance, buttermilk might be found eminently appropriate.—*Ther. Gazette.*

